# Comparative study on the effects of meltwater and various other water sources on plant growth

**DOI:** 10.3389/fpls.2026.1785205

**Published:** 2026-05-15

**Authors:** Shiwen Wang, Fan Zhang, Lijuan Zhang, Yifeng Wang

**Affiliations:** 1 Heilongjiang Province Key Laboratory of Geographical Environment Monitoring and Spatial Information Service in Cold Regions, Harbin Normal University, Harbin, Heilongjiang, China; 2 College of Business Studies, China Academy of Cultural and Tourism Science and Technology Innovation, Shaoxing University, Shaoxing, Zhejiang, China

**Keywords:** fresh weight, mechanisms of influence, plant growth, snowmelt water, stem diameter

## Abstract

**Introduction:**

Snowmelt water as recharge water has a significant effect on plant growth, but quantitative evidence to support this issue is lacking. We selected four control water sources, including rainwater, groundwater, river water, and tap water, and comparatively analyzed the quantitative effects of snowmelt water on typical plants by setting up replicated experiments.We also explored the mechanisms of snowmelt water effects on plant growth.

**Methods:**

Four plant species—Cosmos bipinnatus, Lactuca sativa L., Solanum lycopersicum L. and Poa pratensis L. were selected for a controlled experiment in which they were irrigated throughout their entire growth cycle using meltwater and four other water sources (rainwater, groundwater, river water and tap water). The results were analysed comparatively to provide scientific evidence of the quantitative beneficial effects of meltwater on plant growth.

**Results:**

(1) Under controlled conditions, meltwater significantly promoted the growth of all tested plant species, as evidenced by increases in plant height, root length, stem diameter and fresh weight. (2) Snowmelt water promoted plant growth about 46.58% more than plants irrigated with rainfall, groundwater, Songhua River water, and tap water. The most significant effect on the length of the plant root system could be increased by approximately 2.37 times compared with rainfall and approximately 4.11 times compared with tap water. The boosting effect of snowmelt water on plants was most pronounced during their growth spurt. (3) Mechanistic analysis showed that the mechanism by which snowmelt water significantly promoted plant growth was primarily because of a significant reduction in the content of toxicological indicators in snowmelt water.

**Discussion:**

These findings quantify the role of meltwater in promoting plant growth. They confirm the differences in the effects of meltwater and other water sources on plant growth, and the conclusions support efforts to improve the utilisation of meltwater resources. However, the underlying mechanisms by which meltwater influences plant growth require further elucidation and investigation.

## 1 Introduction

Snow, as the main form of wet deposition, has an important effect on the physiological characteristics and ecology of plants (Caron et al., 2025). Therefore, exploring the roles, patterns, and mechanisms between snow cover and ecosystems has been a popular research topic in the field of snow cover ecology. Currently, more than 60% of the world’s landmass is covered by seasonal snow, with the maximum snow extent on land in the northern hemisphere being about 47 × 10^6^ km (Li et al., 2025). Snowmelt water from the melting of winter snow can both replenish soil moisture, providing needed water and nutrients to spring-sown crops, and replenish groundwater sources during dry periods (Gamon et al., 2013; Ide and Oguma, 2013; Kevin et al., 2020). In agriculture, snow is used passively to insulate the soil in the winter and to recharge the water supply in the spring (Legault and Cusa, 2015; Kunishima and Yoshimura, 2025). There is no effective management of snow, let alone any proactive direct use of snowmelt water, which is instead left to naturally accumulate, melt, and form snowmelt runoff. Although snowmelt runoff rehydrates the soil, it also leads to the loss of nutrients (Wheeler et al., 2014; Klein et al., 2018; Martin et al., 2018). Furthermore, snowmelt erosion damages soil fertility in the most valuable agricultural lands (Bard et al., 2015; Meeks and Hunkeler, 2015; Powers et al., 2025). Due to the scouring effects of surface runoff and loam as they flow to converge with rivers, lakes, and groundwater, pollution problems that cannot be ignored have arisen (Carbognani et al., 2014; Pavlovskii et al., 2018). Therefore, the scientific treatment of snowmelt water during the snow-melting period and the rational use of snowmelt water hold great significance for horticultural crop production. It is important to distinguish between two contexts in which snowmelt interacts with agricultural systems. First, as a natural hydrological event, rapid snowmelt generates overland flow that can detach soil particles and transport dissolved nutrients, leading to erosion and fertility loss (Petraglia et al., 2014; Qiu et al., 2016; Sootharet al., 2021). This occurs primarily when melt rates exceed soil infiltration capacity on sloped, bare, or frozen ground. Second, as a managed water resource, collected snowmelt applied via irrigation represents a different scenario: slow, controlled application allows infiltration and nutrient delivery to the rhizosphere, potentially benefiting plant growth (Eltarabily et al., 2020). The present study focuses exclusively on the latter context.

Globally, the amount of freshwater obtained from snowpack on land is about 59,500 × 10^8^ m^3^ per year and is an important part of the hydrological cycle (Yano et al., 2015). Melting snow is also a natural yield enhancer that fertilizes fields, strengthens seedlings, and increases yields (Kawai and Kudo, 2018). In recent years, scholars have conducted snow immersion experiments and found that the use of snow immersion, fast seed germination, and long crops are cold hardy and experience increased yields (Carbognani et al., 2012; Yakutina et al., 2015; Briceño et al., 2015; Rahul and Adhikari, 2023). It has been found that, if snow water is used to soak seed early rice, the catalytic grain buds are long and the root is long. When snow water is used to soak the seeds of trees, tomatoes, cucumbers, cotton, and other plants, the germination time can be advanced by 2 to 3 days, which significantly improves the cold hardiness and disease resistance of the seedlings for a period of time after they emerge from the soil (Wheeler et al., 2015). The germination potential and germination rate of the seeds soaked in snow water were higher than those soaked in well water, which also provided some degree of yield increase, generally in the range of 4–12% (Sedlacek et al., 2014). In addition, some scholars reached the conclusion that watering plants with snow water for a long period of time promoted plant growth and increased yield (He et al., 2025; Julitta et al., 2014; Sedlacek et al., 2016).

Although some research conclusions have been reached regarding seed soaking in snowmelt water and irrigation with snowmelt water. These findings remain largely qualitative in nature, lacking scientific experimental support and quantitative conclusions. Snowmelt water samples were collected in Heilongjiang Province, Northeast China (45°N–50°N), a region characterized by prolonged winter snow cover and seasonal snowmelt that serves as a potential water source for local agriculture. Sampling locations were selected in rural areas, distant from industrial and urban pollution sources, to minimize anthropogenic contamination. This region was chosen because its cold temperate continental climate and snowpack properties are representative of high-latitude agricultural zones where snowmelt irrigation is of practical interest. Despite the previous conclusions on snowmelt seed dipping and snowmelt watering, they basically remain at the qualitative stage, lacking scientific experimental support and quantitative conclusions. In this study, we selected snowmelt water and rainwater, groundwater, river water, and tap water as the experimental water sources. We conducted four typical plant growth observation experiments on four types of plants, namely, cosmos, lettuce, tomato, and dryland morning glory. We clarified the quantitative effects of snowmelt water versus other water sources on plant growth and investigated the mechanisms by which snowmelt water affected plant growth.

## 2 Materials and methods

### 2.1 Experimental design

This study selected Harbin, a representative city in Northeast China, for water source sampling (125°42′-130°10′E, 44°04’-46°40′N), the region exhibits a temperate continental monsoon climate (Zhang et al., 2023). This study selected four representative plant species, namely Cosmos bipinnatus, Lactuca sativa L., Solanum lycopersicum L., and Poa pratensis L. These plants were chosen due to their widespread cultivation, ease of observation, and sensitivity to water quality changes, making them ideal for assessing the impact of different water sources on plant growth. The experimental design involved setting up four treatment groups for each plant species, with each group receiving a different water source: snowmelt water, rainwater, groundwater, river water, and tap water. Each treatment group was replicated three times to ensure statistical reliability. The plants were grown under controlled environmental conditions, including consistent temperature, humidity, and light intensity, to minimize external variables. The growth parameters measured included plant height, root length, stem diameter, and fresh weight.

#### 2.1.1 Experimental water preparation

The experimental water used included snowmelt water, rainfall water, groundwater, Songhua River water, and tap water, which were collected as follows.

- Snowmelt water (hereafter referred to as snow water): This study was conducted on November 19, 2023; November 30, 2023; December 16, 2023; January 7, 2024; and January 21, 2024. Considering the accumulation of pollutants in snow, collection times were evenly distributed across the initial snowfall period, stable period, and melting period. At the end of each snowfall, we collected fresh snow with a snow shovel and placed it in a sterile bucket. After transporting it back to the laboratory, when it melted into snow water, we mixed it all together and collected it into new sterile buckets for storage. We also collected snow on the roof deck of the Third Polytechnic Building of Harbin Normal University.
- Rainfall water (hereinafter referred to as rainwater): heavy rain, thunderstorms, and showers fell in Harbin on July 7, 2023; August 5, 2023; and August 15, 2023, respectively. According to the weather forecast, we arranged and distilled the water-catching containers outside and tightly attached sterilized plastic film to the inner and bottom walls of the containers. After rainfall water collection, we transferred the samples to the laboratory, poured the rainfall into the same sterile bucket for mixing, and stored the sample for later use. We collected the rainfall on the roof terrace of the Polytechnic III building of Harbin Normal University.
- Groundwater: We used Harbin underground well water as the groundwater from the wells near the campus of Harbin Normal University at 45°52′21″N, and 126°30′46″E. We used a pressurized water pump to press out the underground well water, collected it into sterile buckets, and transported it back to the laboratory for storage and spare parts.
- Songhua River water (hereafter referred to as river water): In the nearest section of the Songhua River to Harbin Normal University, we collected samples on June 13, 2023, and December 15, 2023. Twice in the winter and the summer, we collected and distilled river water from the containers and attached sterilized plastic film tightly to the inner and bottom walls of the containers. After transporting it to the laboratory, we poured and mixed the sample into the same sterile bucket and stored it for later use.
- Tap water: In the laboratory of the third building of the Harbin Normal University Polytechnic, we collected the building’s piped tap water two times, 5 days apart. We placed the containers in daylight to dry and dechlorinate, and then transported the containers back to the laboratory, poured and mixed the samples into the same sterile barrel, and stored the samples for future use. Tap water was dechlorinated by exposure to natural daylight in transparent 10-L glass carboys (surface-to-volume ratio: 0.15 cm^-1^) for 72 hours. Containers were placed on a rooftop with unobstructed sunlight (photosynthetically active radiation: 900–1500 μmol·m^-2^·s^-1^) and agitated twice daily. This duration and container type were selected based on standard protocols for chlorine dissipation in horticultural research.

#### 2.1.2 Experimental procedure

This study employed a controlled experimental design, with each treatment group receiving a specific type of experimental water. The plants were carefully transferred into pots filled with the corresponding water types, ensuring that the root systems were fully submerged. Throughout the experimental period, the pots were regularly rotated to ensure uniform exposure to light and other environmental factors. Daily observations were made to record any visible changes in plant morphology or health. To ensure the validity and comparability of the test results, we adopted soilless culture technology, using snowmelt water, rainwater, groundwater, river water, and tap water as the only sources of plant nutrients. To avoid the influence of other factors on plant growth, we kept all other environmental factors the same. The experimental process was as follows.

Step 1. Seeds were screened to eliminate problematic seeds and any genetic differences between individual seeds to ensure high and consistent seed quality. We rotated the sieve to initially remove unsaturated seeds and other debris, followed by using the fan wind to further remove the lighter bad seeds and other debris; then, we removed the seeds and impurities floating on the surface by placing them in clear water and taking the lower layer of precipitated seeds and drying them. The experiment followed a completely randomized design with five water sources and four plant species. For each species × treatment combination, two replicate seedling trays (30 cm × 40 cm) were used, with each tray containing 20 seeds planted at equal spacing. This resulted in a total of 4 species × 5 treatments × 2 trays × 20 plants = 800 individual plants.

**Figure 1:**
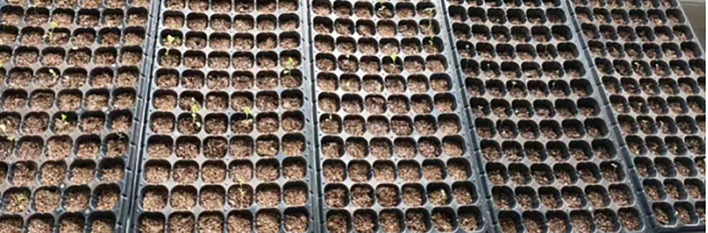
Single-group arrangement of experimental plants.

Step 2. During seeding, screened seeds of each plant were planted in seedling trays (**Figure 1**) with 72 holes per tray and one seed per hole. We repeated two seedling trays for each seed. The seeds were covered with vermiculite at a height of 3/4 of the height in the holes of the seedling trays. We injected 20 mL of snow, rain, groundwater, river water, and tap water into each culture hole using a syringe. The water was replenished every 3 days with 20 mL of water in each hole. The breeding was considered complete when three-quarters of the seeds in the culture tray sprouted.

Step 3. Transplanting was carried out to observe the developmental period and the corresponding indicators of the developmental period. Nine plants with similar growth were selected from the seedling trays and transplanted into three culture pots, i.e., three plants in each pot; the process was repeated three times. The total number of four plants was 180 pots (9 × 4 × 5 pots) with a total of 540 plants (3 × 3 × 3 × 4 × 5 plants). During transplanting, we used a clean iron spoon to separate the plants attached to the vermiculite, followed by removing the plants intact and transplanting them evenly into the culture pots, which were then covered with vermiculite up to three-fifths of the height of the pots. We used a syringe to inject 50 mL of snow, rain, groundwater, river water, and tap water into each of the pots along the edge of the pots and repeated this four times for a total injection volume of 200 mL. The supplemental watering was repeated at 7-day intervals. We continued the whole experiment until all the culture pot plants showed leaf loss and yellowing of new leaves.

Step 4. The remaining plants were transplanted and maintained in the culture. We randomly selected three plants at 7-day intervals to determine the appropriate indexes. For each species × treatment combination, destructive sampling was performed at 7-day intervals (Days 7, 14, 21, 28, and 35 after germination). At each time point, three plants were randomly selected from each of the two replicate seedling trays (i.e., 6 plants total per treatment per time point). Sampled plants were immediately measured for root length, shoot height, and fresh weight, then oven-dried for biomass determination.

### 2.2 Methods

#### 2.2.1 Selection and measurement of indicators

For the measurement of plant growth indexes, because we used vermiculite for soilless culture throughout the experiment, we excluded influencing factors other than water. The water cannot provide all the necessary nutrients to complete the entire plant growth cycle. Thus, the experimental plants could complete only nutrient growth—roots, stems, and leaves. Therefore, after the plant entered the seedling stage, until the beginning of yellow leaves, and the phenomenon of stiff seedlings, which is called the growth period, through successive death to all death, which is called the end of the growth period. This study divided the experimental groups into X1, X2, and X3 based on different stages of plant growth. These represent the early seedling stage, the seedling stage, and the vigorous growth stage, respectively. Developmental stages were defined based on true leaf counts following established criteria (citation). For tomato (Solanum lycopersicum), the early seedling stage comprised plants with 1-3 true leaves (approximately Days 10-20 after sowing), the late seedling stage comprised plants with 4-6 true leaves (Days 20-30), and the rapid vegetative stage comprised plants with >6 true leaves (Days 30-40). For lettuce (Lactuca sativa), corresponding leaf counts were adjusted for its rosette growth habit: early seedling (2-4 leaves), late seedling (5-8 leaves), and rapid vegetative (9+ leaves).

**Figure 2:**
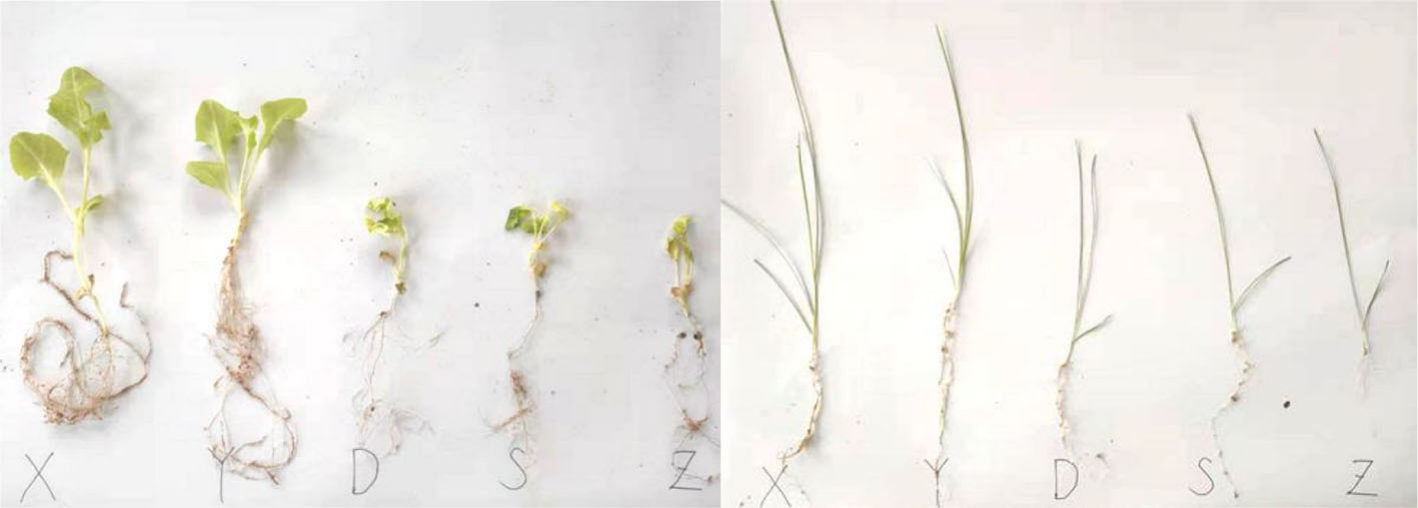
Comparison of plants in different moisture groups.

The measurement indicators were as follows. At the germination (seed sprouting), seedling (cotyledon leaves fully expanding), and vigorous-growth stages, the plant and its root system were dug out completely in a clean vessel, and the vermiculite attached to the root system was cleaned, followed by removing the liquid using a filter paper to keep the plant in its natural state; the process was conducted on a clean tabletop. Plant height is measured from the root collar to the apical meristem (for rosette species such as lettuce) or the base of the youngest fully expanded leaf (for erect species such as tomato). The root collar is identified by gently removing surface substrate until the transition in color and texture between root and stem tissue becomes visible. Using electronic vernier calipers with an accuracy of 0.01 mm, the length from the root collar to the tip of the apical leaf was measured three times, and the average of the three results was taken as the plant height. During the seedling and growth stages, using electronic vernier calipers with an accuracy of 0.01 mm, the length of the diameter above the root collar and in the mid-axis part of the plant body was measured three times, and the average of the three results was taken as the stem diameter of the plant. Next, using an electronic balance with an accuracy of 0.001 g, we measured the weight of the plant when the plant was in a fresh and inactivated state three times and took the average of the three results as the fresh weight of the plant. Furthermore, during the seedling and vigorous-growth stages, we measured the length of the root tip from the root collar to the tip of the main root using an electronic vernier caliper with an accuracy of 0.01 mm. The measurements were taken three times and the average of the three results was taken as root length. This study uses X, Y, D, S, and Z to represent the snowmelt water, rainwater, river water, groundwater, and tap water groups, respectively (**Figure 2**).

#### 2.2.2 Measurement of plant indicators

To exclude the influence of factors other than moisture, we used vermiculite for soilless culture throughout the experiment. Because the nutrients provided by moisture were limited, the experimental plants could complete only the nutrient growth stage (i.e., roots, stems, and leaves).

We divided the plant development period into germination (first leaf appeared), seedling stage (cotyledons fully unfolded after germination), vigorous-growth stage (yellow leaves began to appear, stippling phenomenon), and the end of growth stage (all plants died). We took measurements on plant height (germination, seedling, and vigorous stage), stem diameter (seedling and vigorous stage), fresh weight (germination, seedling, and vigorous stage), root length (seedling and vigorous stage), and number of roots (seedling and vigorous stage), as follows:

We completely dug out the plants and their root systems using clean utensils (one plant was pulled out from each pot, for three plants in total) and cleaned up the vermiculite attached to the root system of the plant. We removed the liquid attached to the plant itself using filter paper, leaving the plant in its natural state on a clean tabletop. We determined plant height from the root collar to the tip of the apical leaf using electronic vernier calipers with an accuracy of 0.01 mm. We determined stem diameter from the length of the diameter of the mid-axis portion of the plant above the root collar using electronic vernier calipers with an accuracy of 0.01 mm. We determined the weight of the plant when the plant was in a fresh and inactivated state using an electronic balance with an accuracy of 0.001 g. We determined the weight of the plant when the plant was in a fresh and inactivated state using electronic vernier calipers with an accuracy of 0.01 mm. We determined root length from the root collar to the tip of the main root of the plant using an electronic vernier caliper with an accuracy of 0.01 mm. We counted all the roots of the plant on a clean tabletop, and the count was taken as the number of roots of the plant.

#### 2.2.3 Water quality monitoring methods

We obtained samples from the experimental water for the determination of 14 water quality indicators in snowmelt, rainfall, groundwater, Songhua River water, and tap water, including total phosphorus, total nitrogen, suspended solids (SS), biochemical oxygen demand (BOD_5_), chemical oxygen demand (COD), hardness, hexavalent chromium, cyanide, sulfide, fluoride, mercury, arsenic, heavy water, and total salt content. Harbin Ante Environmental Testing Co., Ltd. measured all testing indicators except heavy water, and the Beijing Sinochem Institute measured heavy water. The monitoring items and methods are shown in **Table 1**.

**Table 1:** Water quality monitoring methods.

Project	Standard method name and code
pH	Determination of water pH value Glass electrode method GB/T 6920-1986
Chemical oxygen demand	Water quality determination of chemical oxygen demand Dichromate method HJ 828-2017
Suspension	Water quality determination of suspended solids by weight GB/T11901-1989
Five-day BOD	Water quality determination of five-day biochemical oxygen demand Dilution and inoculation method HJ505-2009
Total phosphorus	Water quality determination of total phosphorus ammonium molybdate spectrophotometric method GB/11893-1989
Total nitrogen	Determination of total nitrogen in water alkaline potassium persulfate digestion ultraviolet spectrophotometric method HJ636-2012
Durometer	Water quality determination of total calcium and magnesium EDTA titration method GB/T 7477-1987
Total salt content	Water quality determination of total salt content by weight HJ/T51-1999
Hexavalent chromium	Determination of hexavalent chromium in water Dibenzoyl dihydrazide photometric method GB/T5750.6-2006
Cyanide	Water quality cyanide isonicotinic acid-pyrazolone spectrophotometric method GB/T5750.5-2006
Sulfide	Water quality methylene blue sulfide spectrophotometric method GB/T 16489-1996
Mercury	Water quality mercury atomic fluorescence photometry SL327.2-2005
Arsenic	Water quality arsenic silver diethyldithiocarbamate spectrophotometric method GB/T 7485-1987
Fluoride	Water quality fluoride ion selective electrode method GB/T5750.5-2006
Heavy water (ppm)	Heavy water Fourier infrared spectroscopy

## 3 Results

### 3.1 Effects of snowmelt water on plant growth and development

#### 3.1.1 Comparison of the effects of different water sources on plant developmental indicators

The effects of five culture water sources, namely snow-water, rainwater, groundwater, river-water, and tap-water, on the developmental stages of different plants are shown in **Table 2**. For the four plants (i.e., lettuce, ball tomato, cosmos, and dryland early maturity grass), the time to reach the germination stage, seedling stage, and the peak-growth stage was the earliest for the snow-water group, followed by the rainwater group, the groundwater group, the river-water group, and the tap-water group. The snow-water group reached the end-of-growth stage the latest, indicating that the snow-water group had the longest plant life span, followed by the rainwater group, groundwater group, river-water group, and tap-water group. Analysis of variance (ANOVA) results showed that the snow-water group was significantly different from all other control-water groups (P < 0.05).

**Table 2:** Differences in the developmental stages of the five water source groups.

Plant name	Developmental stage	Snowmelt water	Rainwater	Groundwater	Songhua River water	Tap water
Cosmos bipinnatus	Germination period	July 21	+5^b^	+6^b^	+7^b^	+11^a^
	Seedling stage	July 29	+6^c^	+11^b^	+12^b^	+21^a^
	Vigorous growth period	August 17	+10^b^	+17^a^	+18^a^	+17^a^
	End of growth	October 18	−4^b^	−9^c^	−8^c^	−14^d^
Lactuca Sativa L.	Germination period	July 20	+4^b^	+4^b^	+4^b^	+4^a^
	Seedling stage	July 27	+6^c^	+8^b^	+9^b^	+15^a^
	Vigorous growth period	September 3	+6^b^	+18^a^	+20^a^	+7^b^
	End of growth	October 21	−2^b^	−14^c^	−13^c^	−19^c^
Solanum lycopersicum L.	Germination period	July 17	+6^b^	+7^b^	+7^b^	+10^a^
	Seedling stage	July 20	+18^c^	+22^b^	+22^b^	+31^a^
	Vigorous growth period	August 11	+8^c^	+25^b^	+25^b^	+29^a^
	End of growth	October 19	−5^b^	−11^c^	−11^c^	−16^d^
Poa pratensis L.	Germination period	July 22	+4^b^	+5^b^	+5^b^	+10^a^
	Seedling Stage	August 4	+5^c^	+9^b^	+10^b^	+20^a^
	Vigorous growth period	September 7	+5^c^	+13^a^	+13^a^	+9^b^
	End of growth	October 20	−4^b^	−11^c^	−9^c^	−14^d^

Different lowercase letters in the same column indicate significant differences at the 0.05 level.

The effect of five culture water sources on the plant height of different plants is shown in **Table 3**. The results showed that for all four plants (i.e., lettuce, ball tomato, cosmos, and morning glory), the plant heights to reach early seedling stage, seedling, and peak-growth stages were the largest in the snow-water group, and the others in the order of rainwater, groundwater, river-water, and tap-water groups. The results of ANOVA showed that the snow-water group was significantly different from the control-water group (P < 0.05). The height of plants irrigated with snowmelt was about 2.85 times higher than that of plants irrigated with tap water. The effect of snowmelt water on plant height was most pronounced at the peak production stage, when it was about 3.4 times higher than that of plants irrigated with tap water, and at the germination and seedling stages, when the effect was of similar magnitude. The effect of snowmelt water on lettuce was the most significant at the growth stage, with plant heights up to 5.77 times higher than those irrigated with tap water.

**Table 3:** Differences in plant height of different plants under five water sources (mm).

Plant name	Developmental stage	Snowmelt water	Rainwater	Groundwater	Songhua River water	Tap water
Lactuca sativa L. var. Ramosa Hort.	Early seedling stage	67.61^a^	53.53^b^	40.40^c^	31.57^cd^	22.48^d^
	Seedling stage	223.14^a^	181.17^b^	121.69^c^	111.84^c^	61.29^d^
	Vigorous growth period	367.71^a^	263.92^b^	192.07^c^	182.12^c^	63.66^d^
Lycopersicon esculentum Mill	Early seedling stage	57.45^a^	38.89^b^	31.29^c^	30.05^c^	27.13^c^
	Seedling stage	128.21^a^	106.92^b^	97.33^c^	95.14^c^	81.29^d^
	Vigorous growth period	337.50^a^	289.94^b^	210.84^c^	192.49^c^	128.05^d^
Cosmos bipinnata Cav. bipinnata Cav.	Early seedling stage	103.32^a^	70.64^b^	54.13^c^	53.20^c^	38.11^d^
	Seedling stage	268.18^a^	206.00^b^	112.23^c^	110.44^c^	86.30^d^
	Vigorous growth period	454.50^a^	404.94^b^	295.84^c^	277.49^c^	185.05^d^
Poa pratensis L.	Early seedling stage	117.18^a^	85.15^b^	60.70^c^	58.17^c^	52.40^c^
	Seedling stage	198.82^a^	140.00^b^	116.59^c^	98.27^d^	96.73^d^
	Vigorous-growth period	331.86^a^	242.77^b^	168.01^c^	126.32^d^	117.70^d^

Different lowercase letters in the same column indicate significant differences at the 0.05 level.

**Figure 3:**
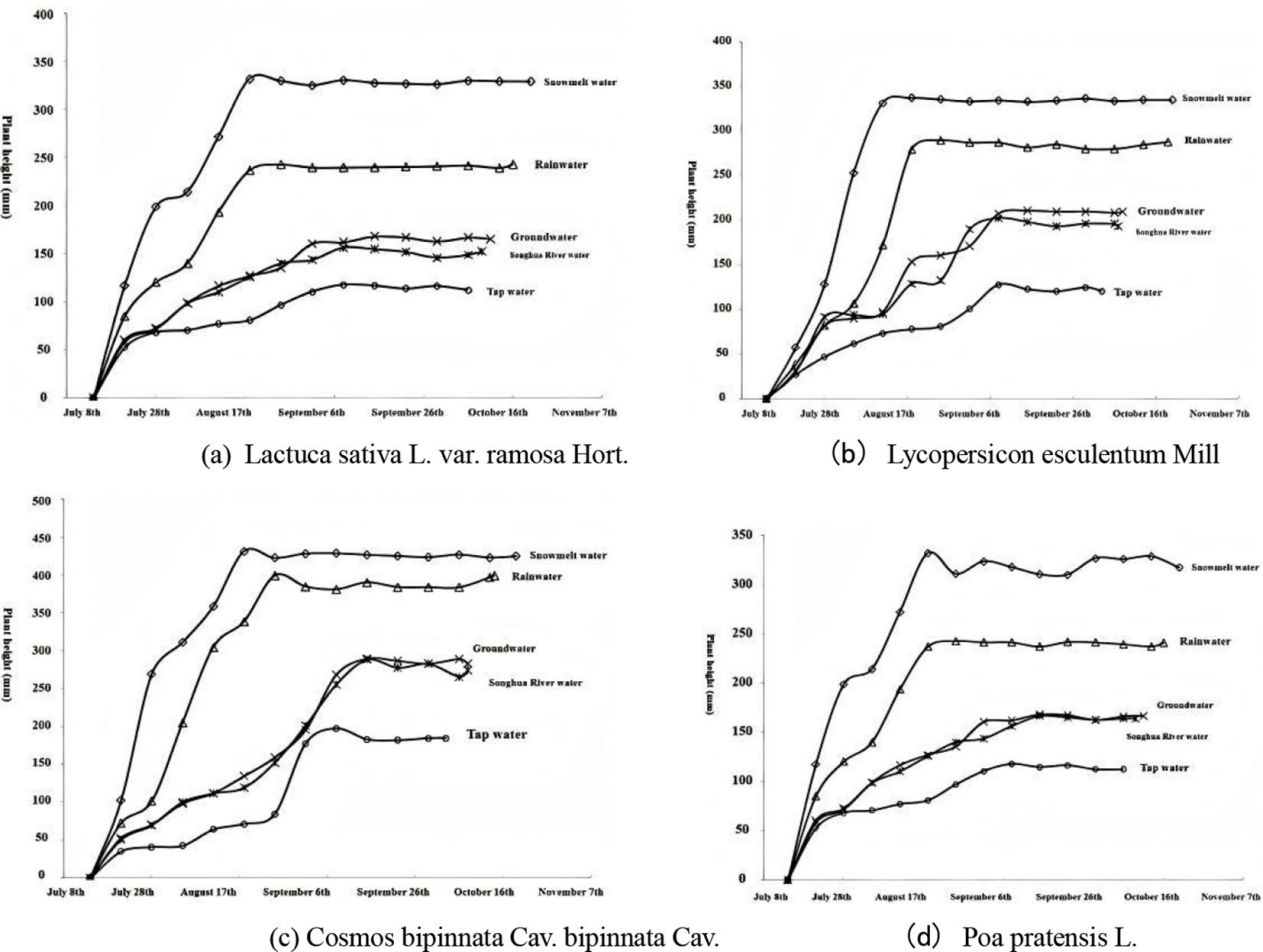
Cumulative growth curves for plant height in four species. **(a)** Lactuca sativa L. **(b)** Lycopersicon esculentum Mill. **(c)** Cosmos bipinnatus. **(d)** Poa pratensis L.

The cumulative growth curves of plant heights of different plants under five culture water sources are shown in **Figure 3**. It can be seen that the daily growth rates of plant heights of the four plants reaching the peak-growth period were the snow-water group > rainwater group > groundwater group > river-water group > tap-water group. In the case of lettuce, for example, the growth rate of the snow water group to reach the peak was 1.71, 3.00, 3.28 and 10.72 times higher than that of the rainwater, groundwater, river-water, and tap-water groups, respectively.

### 3.2 Comparison of the effects of different water sources on plant growth indicators

The effect of five culture water sources on different stem diameter, fresh weight, root length and root number indices is shown in **Table 3**. The results showed that for the lettuce, ball tomato, cosmos, and morning glory, the indicators of stem diameter, fresh weight, root length, and root number were greatest in the snow-water group, followed by the rainwater group, groundwater group, river-water group, and tap-water group, which showed consistent characteristics at different reproductive periods. ANOVA results consistently showed (**Table 4**) that for all four plants (i.e., lettuce, Solanum lycopersicum L., cosmos, and morning glory), the snowmelt-water group was significantly different from all control-water groups (P < 0.05).

**Table 4:** Effects of water sources on stem diameter, fresh weight, root length, and root number of different plants.

Plant growth indicators	Plant name	Developmental stage	Snowmelt water	Rainwater	Groundwater	Songhua River water	Tap water
Stem diameter (mm)	Lactuca sativa L. var. Ramosa Hort.	Seedling stage	1.53^a^	1.28^b^	0.85^c^	0.85^c^	0.63^d^
		Vigorous growth period	2.64^a^	2.18^b^	1.40^c^	1.37^c^	0.99^d^
	Lycopersicon esculentum Mill	Seedling stage	1.38^a^	0.85^b^	0.68^c^	0.57^c^	0.49^c^
		Vigorous growth period	2.03^a^	1.77^b^	1.57^c^	1.54^c^	1.20^d^
	Cosmos bipinnata Cav. bipinnata Cav.	Seedling stage	1.40^a^	0.88^b^	0.67^c^	0.65^c^	0.48^d^
		Vigorous growth period	3.17^a^	2.18^b^	1.60^c^	1.57^c^	1.21^d^
	Poa pratensis L.	Seedling stage	0.90^a^	0.61^b^	0.31^c^	0.29^c^	0.16^d^
		Vigorous growth period	2.08^a^	1.37^b^	0.86^c^	0.83^c^	0.36^d^
Fresh weight (mg)	Lactuca sativa L. var. Ramosa Hort.	Germination stage	0.543^a^	0.276^b^	0.176^c^	0.147^c^	0.035^d^
		Seedling stage	0.783^a^	0.461^b^	0.267^c^	0.248^c^	0.124^d^
		Vigorous growth period	1.371^a^	0.840^b^	0.530^c^	0.473^c^	0.277^d^
	Lycopersicon esculentum Mill	Germination period	0.291^a^	0.125^b^	0.038^c^	0.032^c^	0.030^c^
		Seedling stage	1.761^a^	1.548^b^	1.291^c^	1.221^c^	0.612^d^
		Vigorous-growth period	2.650^a^	2.054^b^	1.449^c^	1.337^c^	0.964^d^
	Cosmos bipinnata Cav. bipinnata Cav.	Germination period	0.446^a^	0.218^b^	0.191^c^	0.189^c^	0.127^d^
		Seedling stage	2.077^a^	1.300^b^	1.034^c^	0.972^c^	0.777^d^
		Vigorous-growth period	2.874^a^	2.265^b^	1.806^c^	1.682^cd^	1.427^d^
	Poa pratensis L.	Germination period	0.104^a^	0.053^b^	0.025^c^	0.024^c^	0.019^d^
		Seedling stage	0.234^a^	0.176^b^	0.082^c^	0.070^c^	0.035^d^
		Vigorous-growth period	1.200^a^	0.584^b^	0.177^c^	0.158^c^	0.108^d^
Root length (mm)	Lactuca sativa L. var. Ramosa Hort.	Seedling stage	127.29^a^	73.84^b^	51.23^c^	47.99^c^	12.66^d^
		Vigorous-growth period	220.21^a^	137.50^b^	118.73^c^	110.48^c^	73.55^d^
	Lycopersicon esculentum Mill	Seedling stage	110.59^a^	79.57^b^	57.17^c^	55.15^c^	52.69^c^
		Vigorous-growth period	272.14^a^	192.20^b^	177.42^c^	174.20^c^	133.78^d^
	Cosmos bipinnata Cav. bipinnata Cav.	Seedling stage	145.33^a^	112.71^b^	78.25^c^	77.63^c^	73.18^c^
		Vigorous-growth period	290.14^a^	207.20^b^	135.42^c^	133.20^c^	107.78^d^
	Poa pratensis L.	Seedling stage	86.75^a^	65.53^b^	57.86^c^	53.38^cd^	50.07^c^
		Vigorous-growth period	212.84^a^	176.89^b^	139.33^c^	127.22^c^	110.89^d^
Number of roots	Lactuca sativa L. var. Ramosa Hort.	Seedling stage	2^a^	2^a^	2^a^	2^a^	1^b^
		Vigorous-growth period	17^a^	16^a^	14^b^	13^b^	5^c^
	Lycopersicon esculentum Mill	Seedling stage	2^a^	2^a^	2^a^	2^a^	1^b^
		Vigorous-growth period	18^a^	15^b^	15^b^	14^b^	3^c^
	Cosmos bipinnata Cav. bipinnata Cav.	Seedling stage	2^a^	2^a^	2^a^	2^a^	1^b^
		Vigorous-growth period	15^a^	14^a^	5^b^	5^b^	3^c^
	Poa pratensis L.	Seedling stage	2^a^	2^a^	2^a^	2^a^	1^b^
		Vigorous-growth period	4^a^	4^a^	3^a^	3^a^	1^b^

Different lowercase letters in the same column indicate significant differences at the 0.05 level.

#### 3.2.1 Effect of different water sources on plant stem diameter

In the seedling stage of cosmos bipinnatus, the average stem diameter of each plant in treatment groups X_1_ (nowmelt water), Y_1_ (rainwater), D_1_ (river water), S_1_ (groundwater), and Z_1_ (tap water groups) was 1.43 mm, 0.90 mm, 0.69 mm, 0.66 mm, and 0.50 mm, respectively. In the vigorous stage, the average stem diameter of each plant in treatment groups X_2_, Y_2_, D_2_, S_2_, and Z_2_ was 3.24 mm, 2.01 mm, 1.57 mm, 1.56 mm, and 1.34 mm, respectively. It can be seen that the mean stem diameter of a single plant in treatment groups X_1_ and X_2_ with snowmelt water as the sole recharge water was the maximum among all the treatment groups, and the mean stem diameter of a single plant in treatment groups Z_1_ and Z_2_ with tap water as the sole recharge water was the minimum among all the treatment groups. The standard deviation of the single-stem diameter data for each of the treatment groups X_1_, Y_1_, D_1_, S_1_, and Z_1_ is given in **Table 5**. The standard deviation of the average single-stem diameter data of each treatment group was relatively small and the sample variation was relatively smooth. The rainwater treatment group had the largest standard deviation of the single-stem diameter data and fluctuated the most compared with other groups. The tap-water treatment group had the smallest standard deviation of single-stem diameter data, and the sample changes were smoother and fluctuated the least compared with the other treatment groups. The standard deviation of the single-stem diameter data of the other treatment groups did not differ much, and the standard deviation was slightly smaller than that of the tap-water treatment group.

**Table 5:** Mean and standard deviation of stem diameter ().

Category	Average value	Standard deviation								
X_1_	1.43	0.06	a				A			
Y_1_	0.90	0.09		b				B		
D_1_	0.69	0.06			c				C	
S_1_	0.66	0.05			c				C	
Z_1_	0.50	0.02				d				D
X_2_	3.24	0.23	a				A			
Y_2_	2.01	0.25		b				B		
D_2_	1.57	0.14			c				C	
S_2_	1.56	0.15			c				C	
Z_2_	1.34	0.14				d				D

When lettuce was in the seedling stage, the average stem diameters of single plants in treatment groups X_1_, Y_1_, D_1_, S_1_, and Z_1_ were 1.53 mm, 1.28 mm, 0.90 mm, 0.87 mm, and 0.63 mm, respectively; when lettuce was in the vigorous stage, the average stem diameters of single plants in treatment groups X_2_, Y_2_, D_2_, S_2_, and Z_2_ were 2.64 mm, 2.18 mm, 1.40 mm, 1.37 mm, and 0.99 mm, respectively; and when tomato was in the seedling stage, the average stem diameters of single plants in treatment groups X_1_, Y_1_, D_1_, S_1_, and Z_1_ were 2.64 mm, 2.18 mm, 1.40 mm, 1.37 mm, and 0.99 mm, respectively. The average stem diameters of individual plants in X_1_, Y_1_, D_1_, S_1_, and Z_1_ were 1.40 mm, 0.88 mm, 0.67 mm, 0.60 mm, and 0.48 mm, respectively, when tomato was in the seedling stage. The average stem diameter of each treatment group X_2_, Y_2_, D_2_, S_2_, and Z_2_ was 2.17 mm, 1.78 mm, 1.60 mm, 1.58 mm, and 1.21 mm, respectively, which was basically the same as that of cosmos. These results were consistent with those of A. persica. The average stem diameter of a single plant in the treatment group, with snowmelt water as the sole recharge water, was the largest among all the treatment groups. The average stem diameter of a single plant in the treatment group, with tap water as the sole recharge water, was the smallest among all the treatment groups.

During the seedling stage of Solanum lycopersicum L. plants, the average stem diameter per plant in the treatment groups X1, Y1, D1, S1, and Z1 was 1.40 mm, 0.88 mm, 0.67 mm, 0.60 mm, and 0.48 mm, respectively. The standard deviations for stem diameter data in treatment groups X1, Y1, D1, S1, and Z1 were 0.10, 0.14, 0.10, 0.05, and 0.05, respectively. The standard deviations for average stem diameter data across all treatment groups were relatively small, indicating stable sample variation. The standard deviation for stem diameter data in rainwater treatment group Y1 was the largest, exhibiting the greatest fluctuation compared to other groups. At P<0.05 or P<0.01 significance levels, treatment group X2 showed significant differences compared to groups Y2, D2, S2, and Z2. This indicates significant differences between the snowmelt water treatment group and the groundwater treatment group. During the tomato’s vigorous growth period, the average stem diameters per plant for treatment groups X2, Y2, D2, S2, and Z2 were 2.17 mm, 1.78 mm, 1.60 mm, 1.58 mm, and 1.21 mm, respectively. Thus, treatment group X2, which used snowmelt water as the sole water source, exhibited the largest average stem diameter per plant among all treatment groups, while treatment group Z2, which used tap water as the sole water source, exhibited the smallest average stem diameter per plant. The standard deviations for stem diameter data in treatment groups X2, Y2, D2, S2, and Z2 were 0.31, 0.24, 0.21, 0.22, and 0.18, respectively. The standard deviations for average stem diameter data across all treatment groups were relatively small, indicating relatively stable sample variation. The standard deviation for stem diameter data in the snowmelt water treatment group X1 was the largest, exhibiting the greatest fluctuation compared to other groups. In summary, snowmelt water significantly promotes stem diameter growth in tomato seedlings compared to groundwater and other water sources.

The experimental results for the fourth plant species, Poa pratensis L., similarly demonstrated that under conditions of P<0.05 or P<0.01, both snowmelt water treatment groups X1 and X2 exhibited significant differences compared to the control group. This indicates that the snowmelt water treatment groups showed significant differences relative to the groundwater treatment group. Snowmelt water significantly promoted the stem diameter growth of Poa pratensis L.

These results showed that snowmelt water irrigation had a significant effect on the growth of the stem diameter of plants. The growth of the stem diameter of plants irrigated with snowmelt water was about 2.41 times higher than that of plants irrigated with tap water, in which the growth promotion effect of snowmelt water on the growth of plant stem thickness was more noticeable in the peak-growth period of plants.

#### 3.2.2 Effect of different water sources on plant fresh weight

Experimental test results for all four plant species indicate that snowmelt water significantly promotes growth in Cosmos bipinnatus, Lactuca sativa L., Solanum lycopersicum L., and Poa pratensis L. compared to other water sources, both during the seedling stage and the vigorous growth stage. Research findings indicate that snowmelt water significantly affects the fresh weight growth during the seed germination stage. Given the similarity in experimental results across the four plant species, the findings from the cosmos bipinnatus data will be used as an illustrative example.

**Figure 4:**
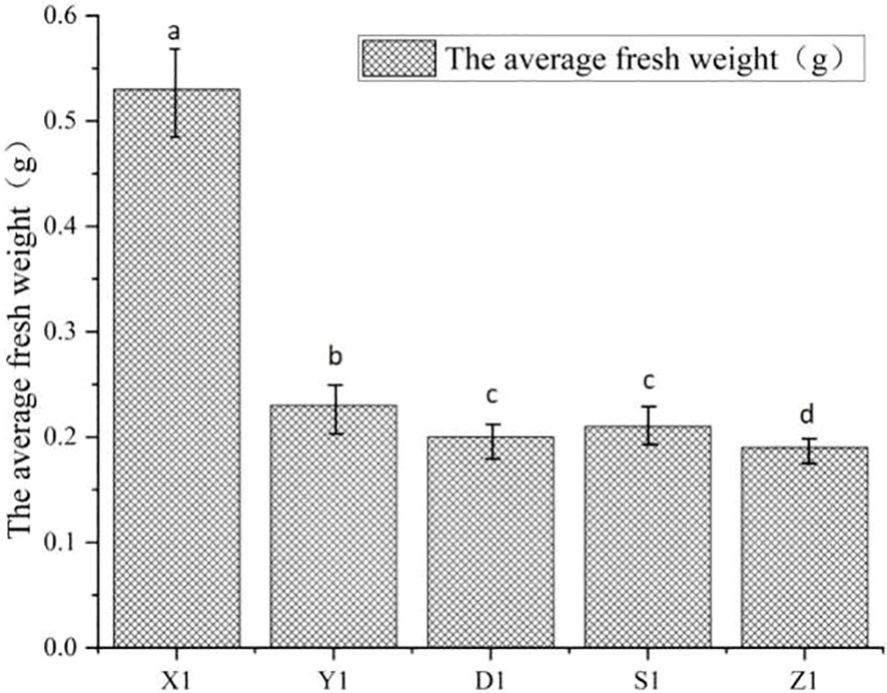
Effect of different supplemental water on fresh weight of cosmopolitan chrysanthemum during germination period.

For example, when cosmos bipinnatus was in the germination stage, the average fresh weight of each single plant in treatment groups X_1_, Y_1_, D_1_, S_1_, and Z_1_ was 0.505 g, 0.239 g, 0.192 g, 0.187 g, and 0.131 g, respectively (**Figure 4**). When cosmos was in the seedling stage, the average fresh weight of each single plant in treatment groups X_2_, Y_2_, D_2_, S_2_, and Z_2_ was 2.075 g, 1.303 g, 1.056 g, 1.000 g, and 0.730 g, respectively. Under the conditions of P < 0.05 or P < 0.01, we observed significant differences between the snowmelt water treatment group X_1_ and the treatment groups Y_1_, D_1_, S_1_, and Z_1_, which indicated that the snowmelt water treatment group was significantly different from the groundwater treatment group. We observed significant differences between the rainwater treatment group Y_1_ and the treatment groups X_1_, D_1_, S_1_, and Z_1._ We also observed significant differences between the groundwater treatment group D_1_ and treatment groups X_1_, Y_1_, and Z_1_. Additionally, the Songhua River water treatment group S_1_ was significantly different from treatment groups X_1_, Y_1_, and Z_1_. Other experimental plants showed similar results.

#### 3.2.3 Effect of different water sources on plant root length

Experimental results indicate that snowmelt water significantly promotes plant root growth compared to other water sources. Taking data from cosmos bipinnatus as an example. The experimental results are shown in **Table 5**. It should be noted that the root growth phenomena observed, in conjunction with their proportional relationship to stem growth and stable tissue water content, indicate a coordinated growth response rather than a stress-induced morphological adaptation. Nevertheless, this study did not directly quantify root structure parameters (such as lateral root density or root length per unit length), which will constitute a direction for future research.

The results of this study showed that the treatment group with snowmelt water as the only recharge water had the largest average plant height per plant among all treatment groups, and the snowmelt water treatment group had the smallest standard deviation data and the smallest fluctuation compared with the other groups. The snowmelt water treatment group had a significant advantage in root length growth compared with the other supplemental water treatment groups for all four plant species at all growth periods in the experiment. Snowmelt water had a significant effect on plant root length compared with other recharge water. In the seedling period of cosmos bipinnatus, the single-plant root lengths and average root lengths of treatment groups X_1_, Y_1_, D_1_, S_1_, and Z_1_ are shown in **Table 6**. During the period in which the cosmos was in the vigorous-growth stage, the average plant height of each single plant in treatment groups X_2_, Y_2_, D_2_, S_2_, and Z_2_ was 293.05 mm, 220.98 mm, 150.86 mm, 147.46 mm, and 119.75 mm, respectively. 

**Table 6:** Root length of portinus in the seedling stage.

Indicator parameters	X_1_	Y_1_	D_1_	S_1_	Z_1_
Plant root length (mm)	143.17	120.45	80.55	76.69	71.20
	140.54	125.10	74.06	79.26	79.04
	146.47	111.19	80.01	77.39	72.96
	148.62	119.07	78.74	73.78	72.28
	151.13	120.33	75.09	75.92	71.99
	140.36	122.94	77.85	79.08	73.60
	165.98	117.78	78.71	77.76	73.77
	161.33	126.00	76.23	74.10	76.85
	150.30	129.77	80.35	78.61	75.01
Average value	149.77	121.40	77.95	76.95	74.08

The daily growth rates of plant stem diameter, fresh weight, root length, and root number were consistently shown to be as follows: snow-water group > rainwater group > groundwater group > river-water group > tap-water group (**Table 7**). The results indicate that snowmelt water demonstrated a superior ability in promoting plant growth across multiple indicators when compared to other water sources. Compared to rainwater, groundwater, river water, and tap water, snowmelt water increased the growth rate of plant stem diameter by 45.77%, 67.25%, 70.42%, and 79.23%, respectively. Compared to tap water, snowmelt water can increase plant fresh weight growth rates by up to 80.29% and boost root length growth by 72.28%.

**Table 7:** Average daily growth rate of plant growth indicators.

Plant growth indicators	Plant name	Snowmelt water	Rainwater	Groundwater	Songhua river water	Tap water
Stem diameter (mm/d)	Lactuca sativa L. var. Ramosa Hort.	0.079	0.049	0.029	0.026	0.018
	Lycopersicon esculentum Mill	0.072	0.035	0.026	0.023^c^	0.017
	Cosmos bipinnata Cav. bipinnata Cav.	0.090	0.047	0.026	0.025	0.018
	Poa pratensis L.	0.043	0.023	0.012	0.012	0.006
Fresh weight (g/d)	Lactuca sativa L. var. Ramosa Hort.	0.044	0.020	0.0104	0.006	0.004
	Lycopersicon esculentum Mill	0.098	0.062	0.0378	0.0352	0.028
	Cosmos bipinnata Cav. bipinnata Cav.	0.113	0.058	0.0370	0.033	0.021
	Poa pratensis L.	0.019	0.008	0.0029	0.003	0.001
Root length (mm/d)	Lactuca sativa L. var. Ramosa Hort.	14.443	6.999	4.503	3.719	3.579
	Lycopersicon esculentum Mill	14.614	8.485	5.935	5.650^c^	4.551
	Cosmos bipinnata Cav. bipinnata Cav.	17.496	10.618	5.943	5.751	4.261
	Poa pratensis L.	11.448	7.387	5.191	4.662	3.688

The results show that the consistent higher daily growth rates observed in the snowmelt water group for stem diameter, fresh weight, root length, and root number suggest that snowmelt water contains beneficial elements or properties that enhance plant physiological processes. This could be attributed to its unique chemical composition, possibly rich in minerals or nutrients that are more readily available or in a form more easily absorbed by plants. Furthermore, the reduced standard deviation in growth parameters within the snowmelt water treatment group implies a more stable and uniform growth environment, which is crucial for optimal plant development. The significant differences noted between the snowmelt water group and other treatment groups, such as groundwater, rainwater, river water, and tap water, underscore the importance of water source selection in horticultural practices. 

### 3.3 Analysis of the mechanism of the effect of snowmelt water on plant growth

In this study, we focused on comparing the water quality characteristics of snowmelt with those of other water sources, to reveal the mechanisms underlying plant growth and development. We analyzed 14 indicators, including total phosphorus, total nitrogen, pH, SS, BOD, COD, hardness, and hexavalent chromium, in the water sources, as shown in **Table 8**. We categorized these indicators as either beneficial or inhibitory to plant growth. The beneficial indicators included total phosphorus and total nitrogen, whereas the inhibitory indicators included toxicological indicators, such as SS, BOD_5_, COD, hardness, hexavalent chromium, cyanide, sulfide, fluoride, mercury, arsenic, heavy metals, and total salinity. 

**Table 8:** Water quality monitoring results of different water sources (mg/L) .

Influence element	Snowmelt water	Rainwater	Groundwater	Songhua River water	Tap water
Total phosphorus	0.108	0.087	0.227	0.285	0.021
Total nitrogen	0.899	0.654	0.994	1.05	0.087
PH	7.302	6.932	6.628	6.460	6.980
SS	14.723	17.126	26.79	24.435	6.398
BOD_5_	≤1	≤1	9.14	19.794	≤1
COD	1.229	1.629	19.361	65.765	1.436
Hardness	69.815	145.755	282.25	799.525	267.328
Hexavalent chromium	0.033	0.048	0.085	0.078	0.048
Cyanide	0.023	0.045	0.165	0.225	0.043
Sulfide	0.148	0.248	0.333	0.430	0.193
Fluoride	0.458	0.893	1.323	1.283	0.928
Mercury	≤0.001	≤0.001	0.002	0.002	≤0.001
Arsenic	≤0.010	≤0.010	0.038	0.045	≤0.010
Heavy water (‰)	0.0012	0.0022	0.0036	0.0038	0.0032
Total salt content	190.25	182	1698	1704.5	428

**Figure 5:**
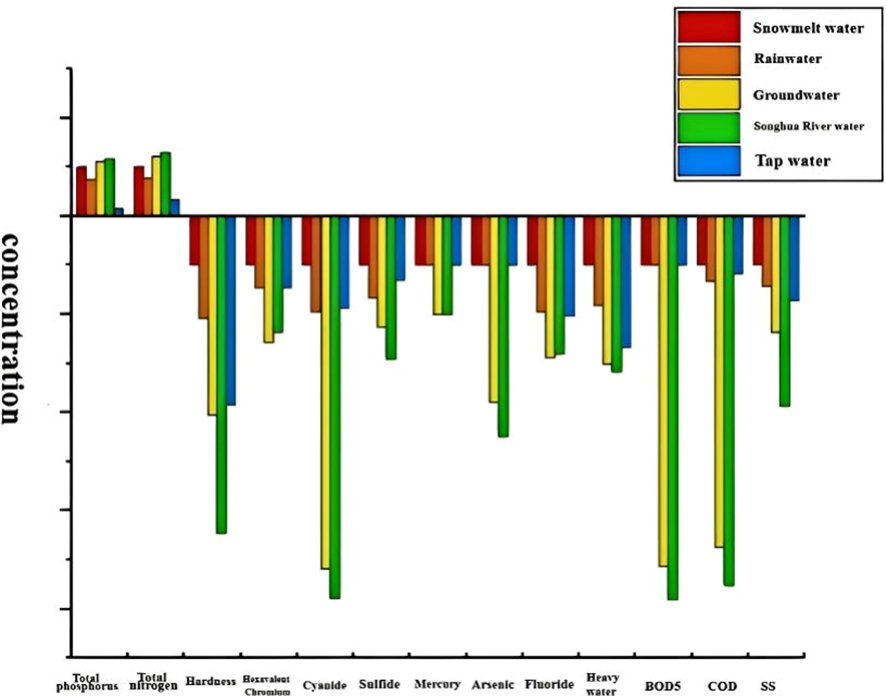
Comparison of pollution content in different water sources.

Hardness represents the high content of calcium and magnesium ions in water samples. Excess calcium and magnesium ions inhibit the absorption of water by plants. Hexavalent chromium leads to nitrification in plants, and nitrification causes local anaerobic environments by depletion of oxygen; cyanide can block aerobic respiration and inhibit plant respiration; most of the sulfides in water are converted to hydrogen sulfide, which is poisonous to plants. Trace fluorine does not have any impact on plant growth, but fluoride accumulates and transfers to the tips and edges of leaves, leading to plants becoming poisonous. Mercury and arsenic are highly toxic, and the higher the concentration of the plant, the stronger the poison. If an organism ingests heavy water, its metabolic rate will be significantly affected, and the ingestion of radioactive heavy water will result in death due to the disruption of biological structures. When the total salt was less than 1,000 mg/L, no inhibition occurred, but more than 1,000 mg/L of salt had an inhibitory effect on the growth of crops, which made it difficult for plants to absorb water and nutrients. Snow water had a lower amount of total salt, which did not have an inhibitory effect on the growth of plants (**Figure 5**).

The results showed that the total phosphorus and total nitrogen in snowmelt water were higher than those in rainwater and tap water but were lower than those in groundwater and river water. The pH value of snowmelt water was higher than that of all control-water sources, and snowmelt water was weakly alkaline, whereas the control water source was weakly acidic. The toxicological indicators of snowmelt water, such as SS, BOD_5_, COD, hardness, hexavalent chromium, cyanide, sulfide, fluoride, mercury, arsenic, and heavy water, were the lowest. The total salt content in snowmelt water was higher than that in rainwater and tap water but lower than that in groundwater and river water. We observed that the content of nutrients in snowmelt water was at a medium level, and the content was not much different from other water sources; snowmelt water was weakly alkaline, within a reasonable range of plant growth needs; snowmelt water in organic pollution was very low, and the toxicity of snowmelt water was small. Additionally, the content of heavy water in snowmelt water was far less than that in other water sources. In conclusion, the mechanism of snowmelt water’s noticeable promotion of plant growth was caused by the significantly low content of toxicity indicators.

## 4 Discussion

Scholars have been conducting plant cultivation work in alpine meadows near glaciers. Because of the high altitude and cold climate, plant growth conditions in this area are usually quite harsh. Previous research, however, has found that the snowmelt from the melting glaciers continuously seeped into the soil, keeping the soil moisture at a relatively suitable level, which allowed the plants to grow to be particularly lush. This natural irrigation method not only conserved water resources but also effectively increased vegetation coverage and ecosystem stability (Tonin et al., 2019). Other researchers have conducted in-depth studies on the impact of snowmelt irrigation on crops from the perspective of horticultural crop production. The results showed that snowmelt, which is rich in trace elements and has a lower mineral content than ordinary groundwater or surface water, was more suitable for irrigation. Through long-term field trials, previous research has found that crops irrigated with snowmelt showed significant improvements in growth rate, leaf color, and fruit quality, particularly in economically important crops that are sensitive to water quality (Genet et al., 2013). Other studies have focused on the field of floriculture and have noted that snowmelt has a distinct promotional effect on the growth of flowers. In greenhouse cultivation, snowmelt has been used as the main source of irrigation water, and the results have shown that the root systems of the flowers were more developed, the colors were more vibrant, and the flowering period was extended. This study provides direct evidence for the utilization of meltwater in horticultural crops. The results indicated that this may have been related to the low-temperature characteristics of snowmelt and its pure water quality, which effectively regulated the soil environment and reduced the occurrence of pests and diseases (Lucieer et al., 2014).

These previous studies have extensively documented the fact that meltwater promotes plant growth. To further validate these observations, in this study, we conducted a systematic water control experiment to quantitatively investigate the enhancing effects of snowmelt on plant growth. We selected various common plant species as research subjects and conducted irrigation using snowmelt and other water sources under identical light, temperature, and soil conditions. By regularly measuring plant height, leaf area, biomass, and other indicators, researchers have found that plants irrigated with snowmelt water exhibited overall superior growth compared with the control group, particularly in terms of water use efficiency and stress tolerance. The results of this study not only provided scientific evidence for plant cultivation in high-altitude regions but also offered new insight and methods for horticultural crop production in high-latitude areas. Additionally, it is important to note that this study was conducted in a soilless system to maximize mechanistic insight. Consequently, the complex interactions between snowmelt water, soil physicochemical properties, and the native microbiome—which could either buffer or amplify the observed effects—were not captured. It is important to note that all species were grown under identical thermal conditions (22/18 °C) to isolate water source effects. However, this uniform environment does not correspond to the thermal optima of all species: tomato (warm-season) typically requires 25-30 °C for maximum growth, while lettuce (cool-season) performs best at 15-20 °C. Consequently, the observed responses may reflect interactions between water source and mild thermal stress in species grown outside their optimal range. Specifically, if snowmelt water has cooling properties due to its lower temperature, it might benefit lettuce by alleviating high-temperature stress, while potentially exacerbating low-temperature stress in tomato. Future studies should examine snowmelt effects under species-specific optimal temperatures to disentangle these interactions.

In addition, past researchers have analyzed the reasons why snowmelt water promotes plant growth more than other water sources. For example, snowmelt water is believed to have low hardness and neutral pH, which is good for plant growth stimulation (Wadgymar et al., 2018). The nitrogen content of snowmelt water is higher than that of other water in the same volume, and the heavy water content of snowmelt water is one-quarter less than that of ordinary water, which is less poisonous, has fewer hazardous substances, and has strong activity, which is more conducive to the absorption of water by plants (Anthony et al., 2019). To date, however, the corresponding studies are qualitative speculation. In this study, we detected not only hardness, PH, nitrogen content, and heavy water content, but also SS, BOD_5_, COD, hexavalent chromium, cyanide, sulfide, fluoride, mercury, arsenic, total salt content, and other toxicological indicators, which provided a quantitative mechanism for the analysis of snowmelt water to promote plant growth. Whilst these findings from controlled environments provide mechanistic insights into the potential benefits of meltwater for horticultural production, extrapolating them to field-scale food security applications requires extensive validation across diverse crops and environmental conditions. This reformulation acknowledges the potential relevance while clearly delineating the limitations of the current evidence.

In this study, we conducted an experiment using the soilless culture technique with vermiculite to exclude the effect of soil nutrients on plant growth. However, the effect of snow water during the later stages of plant growth remains unclear because the plants were limited by the nutrients in the water and failed to complete the entire life cycle. Additionally, the snow cover in Harbin contains relatively high levels of pollutants, a finding that has been confirmed by our team’s existing research (Fan Zhang et al., 2021). Pollutant levels peak during the snowmelt period. Further quantitative research is needed in subsequent studies to investigate the effects of snowmelt water on plant growth at different levels of snow contamination. This study has limitations in investigating the mechanisms by which meltwater affects plant growth. Future research could strengthen the mechanistic interpretation through additional physiological or biochemical measurements.

The plants selected for this experiment had relatively fast growth rates and obvious growth shapes, all of which were annual plants. Thus, it remains uncertain whether the conclusions from this experiment are applicable to perennial plants. A limitation of this study is the use of a fixed irrigation interval for all species without real-time monitoring of substrate moisture or plant water status. Although the chosen volume and frequency were based on published water use data and were conservative enough to prevent severe drought in the highest-using species, we cannot exclude the possibility that species-specific differences in transpiration led to slight variations in water availability. Consequently, some of the observed growth differences may reflect interactions between water quality and subtle differences in plant water status rather than water quality alone. Future studies should employ soil moisture-based irrigation control (e.g., maintaining constant substrate water content via automated systems) to ensure physiological comparability across species. While fixed-interval sampling was used to track temporal dynamics, we verified that plants across treatments were at similar developmental stages at each harvest (based on leaf number counts). This confirms that the observed differences reflect true treatment effects rather than stage-dependent artifacts. Nevertheless, future studies with more divergent growth responses should consider stage-standardized sampling (e.g., harvesting at specific leaf stages) to ensure comparability. In addition, the snowmelt water used in this experiment was a mixture of snowmelt water after several snowfalls, and further investigation is needed to determine whether differences exist in the effects of snowmelt water on plant growth in different periods. The experimental collection of water source sites in Harbin, and whether differences exist in the effects of snowmelt water on plant growth in different areas should also be further investigated. The tap water used in this study was dechlorinated by prolonged sunlight exposure following established protocols. Although previous validation tests confirmed effective chlorine removal (>90% reduction), we did not measure residual chlorine during the experimental period. Therefore, we cannot entirely exclude the possibility that trace chlorine residues influenced plant responses. Future studies should include real-time chlorine monitoring to eliminate this potential confounding factor. Comparative studies across multiple geographical regions with differing atmospheric deposition patterns and climatic conditions are necessary to determine the broader applicability of meltwater as an irrigation resource.

This study was designed as a comparative assessment of five natural water sources on plant growth under standardized controlled conditions. While we measured multiple water quality parameters and plant growth metrics, our experimental approach does not permit definitive mechanistic conclusions about how specific water constituents influence physiological processes. The observed growth differences, while statistically robust, represent phenotypic responses that could arise from multiple interacting factors. For example, poorer performance under river water irrigation could result from suspended solids physically impeding root function, dissolved organic carbon promoting microbial competition, hardness antagonizing nutrient uptake, or unmeasured trace contaminants—or more likely, a combination of these factors. Disentangling these mechanisms would require targeted experiments with synthetic water recipes, isotope tracers, and molecular-level physiological measurements (e.g., gene expression, enzyme assays), which were beyond the scope of the present study. Future research should systematically manipulate individual water quality parameters to establish causal relationships and identify the specific physiological pathways affected.

While we characterized multiple water quality parameters, this study did not include correlation or regression analyses linking these chemical properties to plant growth metrics. Our use of unmodified environmental samples, while ecologically relevant, resulted in natural covariance among parameters that confounds attribution of effects to specific factors. Therefore, our conclusions are restricted to documenting phenotypic differences among water sources under standardized conditions. Future research should build on these findings by systematically manipulating individual water quality parameters (e.g., using synthetic water recipes with controlled suspended solids, hardness, or DOC concentrations) to identify causal relationships and quantify their relative contributions to growth inhibition or promotion. Such studies could employ multiple regression, principal component analysis, or structural equation modeling to disentangle the complex interactions suggested by our results.

## 5 Conclusions

Our study has indicated that snowmelt water exhibits notable promotional effects on plant growth, as evidenced by its favorable water quality indicators. The main conclusions and contributions of the study are summarized in the following two points:

1. The findings of the study confirmed the growth promotion effect of snowmelt water on plant growth. For the growth indexes of the four plants, lettuce, tomato, cosmos, and morning glory, we observed significant differences between the snowmelt-water group and the rainwater group, the groundwater group, the Songhua River–water group, and the tap-water group (P < 0.05), and in general, the snowmelt water could promote the growth of plants by about 46.58% compared with plants irrigated with rainfall water, groundwater, Songhua River water, and tap water. The most significant effect was on plant root length, which could be increased by about 2.37 times compared with rainfall-water irrigation and 4.11 times compared with tap-water irrigation. The next most significant effect was on plant fresh weight, whereas snowmelt water had the least effect on plant stem diameter compared with rainwater. Compared with tap water, snowmelt water could promote the growth of plant stems by about 77.2%.
2. Our analysis showed that the mechanism by which snowmelt water significantly promoted plant growth was mainly because the content of toxicological indicators in snowmelt water was significantly lower. The content of nutrients in snow water was at a medium level, and the content was not much different from that of other water sources. Snow water was weakly alkaline and was within the reasonable range of plant growth requirements. The organic pollution in snow water was very low, and the toxicity of snow water was small. Additionally, the content of heavy water in snow water was much smaller than that in other water sources. While our results provide strong mechanistic evidence for the benefits of snowmelt water on plant physiology, field trials integrating soil-based systems are essential before these findings can be translated into actionable agricultural irrigation practices. Future research should focus on how the soil microbial community mediates the plant’s response to low-temperature snowmelt irrigation.

## Data Availability

The raw data supporting the conclusions of this article will be made available by the authors, without undue reservation.
